# Cervicovaginal specimen biomarkers for early detection of ovarian and endometrial cancer: A review

**DOI:** 10.1002/cam4.70000

**Published:** 2024-07-19

**Authors:** Kevin J. J. Kwinten, Victor A. Lemain, Joanne A. de Hullu, William P. J. Leenders, Miranda P. Steenbeek, Anne M. van Altena, Johanna M. A. Pijnenborg

**Affiliations:** ^1^ Department of Obstetrics and Gynecology Radboud University Medical Center Nijmegen The Netherlands; ^2^ Department of Obstetrics and Gynecology Canisius Wilhelmina Hospital Nijmegen The Netherlands; ^3^ Predica Diagnostics Nijmegen The Netherlands; ^4^ Department of Obstetrics and Gynecology Catharina Hospital Eindhoven Eindhoven The Netherlands

**Keywords:** biomarkers, early detection, endometrial cancer, ovarian cancer

## Abstract

**Background:**

In the last decade, technical innovations have resulted in the development of several minimally invasive diagnostic cancer tools. Within women at high risk of developing ovarian or endometrial cancer (EC) due to hereditary cancer syndrome, there is an urgent need for minimally invasive and patient‐friendly methods to detect ovarian cancer and EC at an early stage.

**Materials and Methods:**

We performed a systematic search of studies using DNA methylation or mutation analysis, microbiome, or proteomics performed on cervicovaginal specimens (smear, swab, or tampon) intended to detect ovarian and EC published until January 2024.

**Results:**

Included studies (*n* = 36) showed high heterogeneity in terms of biomarkers used and outcomes, and only a few studies reported on the detection of biomarkers in high‐risk subgroups.

**Conclusion:**

Based on the findings in this review, DNA methylation techniques seem to be the most promising for detecting ovarian and EC at early stages in the general population. Future validation of cervicovaginal DNA methylation techniques is needed to determine whether this technique might be beneficial in hereditary high‐risk subgroups.

## 1 INTRODUCTION

Early detection of ovarian and endometrial cancer (EC) has been a subject of research for a long time due to their relatively high prevalence in developed countries. Ovarian cancer (OC) is the most lethal type of gynecological cancer and is commonly diagnosed at an advanced stage, whereas EC is generally diagnosed at an early stage and has a more favorable outcome.^1^, ^2^ The incidence of EC is increasing due to obesity, advanced life expectancy, and interestingly this is mainly attributed to the increased number of patients with non‐endometrioid EC, who generally have a poor outcome.^3^, ^4^, ^5^ To date, screening programs for OC and EC in the general population have not been proven to reduce cancer mortality and are therefore not recommended.^6^, ^7^

Women who are at high risk of developing OC or EC, for example due to a hereditary cancer syndrome, may benefit from specific surveillance or risk‐reducing strategies. Women with a *BRCA1* or *BRCA2* pathogenic variant have a cumulative risk of developing OC at the age of 80 years of respectively 44% and 17%.^8^ Surveillance with transvaginal ultrasound and CA‐125 antigen detection in blood samples to detect OC at an early stage in this group has proven to be ineffective.^6^, ^9^, ^10^ Therefore, the only strategy to reduce OC‐related mortality in *BRCA* pathogenic variant carriers is a preventive risk‐reducing bilateral salpingo‐oophorectomy (RRSO) around the age of 40, which can lead to short‐ and long‐term morbidity caused by early surgical menopause.^11^, ^12^

Lynch syndrome is an autosomal dominant hereditary cancer syndrome caused by pathogenic variants in multiple DNA mismatch repair (MMR) genes (*MLH1*, *MSH2*, *MSH6* and *PMS2*) or by a deletion in the *EPCAM* gene resulting in a loss of expression of *MSH2*.^13^ Women with Lynch syndrome are at an increased risk of developing both OC and EC. They have a mutation‐dependent lifetime risk for developing EC between 13% and 71%, compared with 3% in the general population.^14^, ^15^, ^16^, ^17^, ^18^, ^19^, ^20^ Additionally, *MLH1*, *MSH2*, and *MSH6* mutations are associated with an increased lifetime risk of OC depending on the specific mutation between 1% and 38%, compared to approximately 1.5% in the general population.^14^, ^17^, ^21^, ^22^ Women with Lynch syndrome can opt for annual surveillance, including transvaginal ultrasound with subsequent endometrial biopsy, or consider preventive hysterectomy, including RRSO.^23^ However, evidence of the effectiveness of annual endometrial surveillance is contradictory, and the procedure is often painful for these women.^24^

Women with *PTEN* Hamartoma Tumor Syndrome (PHTS), which is caused by mutations in the *PTEN* tumor suppressor gene, have a lifetime risk of developing EC between 19% and 28%.^25^ Surveillance strategies are expert‐opinion based, as no evidence‐based surveillance guidelines are available.^26^

There is a clinical need for simple and patient‐friendly methods to detect OC and EC at early stages. In 2013, Kinde et al. were the first to show that mutations in OC and EC can be detected in DNA from cervical smears, suggesting that cervical smears could be used for OC and EC detection.^27^ Other techniques, including analyses of the microbiome, epigenome, and proteome, followed. In this review, we provide an overview of studies on cervicovaginal specimen biomarkers for the early detection of OC and EC. We aim to identify the tests with the most clinical potential that might have a future role in OC and EC detection in women with a hereditary risk of developing OC and EC.

## 2 MATERIALS AND METHODS

### 2.1 Search strategy

A systematic review according to the Preferred Reporting Items for Systematic Reviews and Meta‐Analyses (PRISMA) guidelines was performed. This review was registered in the International Prospective Register of Systematic Reviews (PROSPERO, number 377155). An electronic literature search for eligible studies was performed in PubMed, Medline and the Cochrane Library. Articles published between January 2010 and January 2024 were included. The following search string was used on the 8th of January 2024: ((Endometrial Neoplasms [MeSH]) OR (ovarian neoplasms [MeSH])) AND (methylation [MeSH]) OR (RNA, neoplasms [MeSH]) OR (DNA, neoplasms [MeSH]) OR (Proteomics [MeSH]) OR (Biomarkers, Tumor [MeSH]) OR (microbiota [MeSH]) AND ((pap [Title/Abstract]) OR (cervical [Title/Abstract]) OR (vaginal [Title/Abstract]) OR (cervicovaginal [Title/Abstract])).

### 2.2 Study selection

Two independently working authors (KK, VL) screened the articles for relevant titles and abstracts and subsequently full texts using the web application Covidence. When no consensus was reached between the two authors, discrepancies were resolved by consulting a third author (JP).

The included articles needed to be available in full text and written in English and had to report on the detection of OC and/or EC using DNA/RNA methylation or mutation, proteomics, or microbiome. The analyzed material needed to originate from cervicovaginal specimens obtained with a smear, swab, or tampon. Only papers with a minimum of 10 patients were included. Articles matching any of the following criteria were excluded from this review: other screening methods, specimens obtained from nonviable organs, case reports, (systematic) reviews, and studies not specifying the number of participants or methods used (defined as unspecified study).

## 3 RESULTS

**FIGURE 1:**
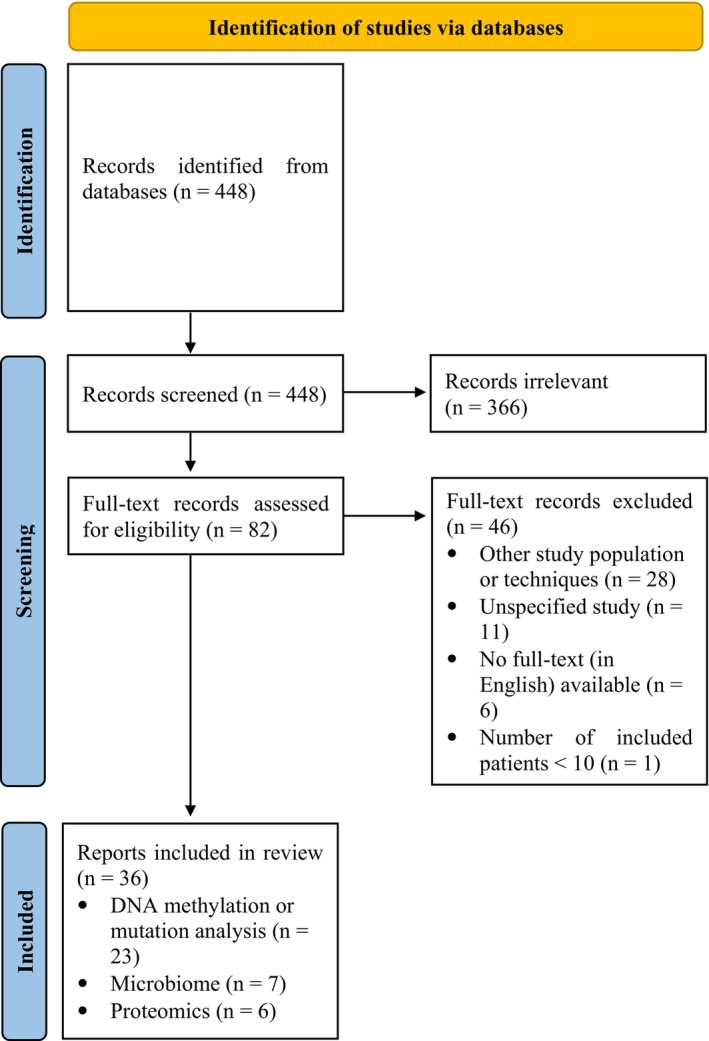
Preferred Reporting Items for Systematic Reviews and Meta‐Analyses flow diagram of the selection of studies to be included in this review.

The search resulted in 448 records, which were all screened on title and abstract. A total of 82 articles were assessed as possibly relevant and were selected for full‐text review. After full‐text review, 46 articles were excluded for the following reasons: other study population or techniques (*n* = 28), unspecified study (*n* = 11), no full‐text (in English) available (*n* = 6) and not enough included patients (*n* = 1). Hence, a total of 36 articles were included in the final review. Twenty‐three studies reported on DNA methylation and/or mutation, seven on the microbiome, and six on proteomics. No studies have reported on RNA methylation. A flowchart of the literature search is shown in Figure 1.

### 3.1 DNA methylation and mutation analysis

A total of 23 studies performed methylation or mutation analysis of DNA isolated from cervicovaginal smears, swabs, or tampons. Twelve studies reported on DNA methylation analysis, nine on DNA mutation analysis, and two on both techniques. An overview of all studies, including the specifications of the techniques and study population, is given in Table 1. The largest study cohorts are briefly summarized below.

**TABLE 1:** Overview of studies using DNA methylation and/or mutation analysis techniques on cervicovaginal specimens to detect OC and EC.

Author, year	Study design	Biospecimen	Tumor type	N total	N OC	N EC	N control	High‐risk profile group	Methodology of testing	Methylated or mutated gene	Findings
Pelegrina, 2023^28^	Case–control	Cervical smear, vaginal swab	EC	246		139	107		NGS	Mutation of *POLE*, *TP53*, *PTEN*, *PIK3CA*, *PIK3R1*, […^*1*^]	Mutational analysis detects 73% of EC in clinical‐collected and self‐collected samples, while specificity was 80% and 90%, respectively
Bakkum‐Gamez, 2023^29^	Case–control	Tampon	EC	192		100	92		qMSP	Methylation of *CDH4*, *c17orf64*, *CYTH2*, *DIDO1*, *EEF1A2*, […^*2*^]	A panel of 28 methylated genes yielded 96% specificity and 82% sensitivity (AUC 0.91). A post‐hoc analysis to a panel of three genes yielded the same AUC as the 28‐panel
Barrett, 2023^30^	Case–control	Cervical smear	EC	493		118	375		Methylation array	Methylation of CpG sites	CpGs methylation identifies EC with a sensitivity and specificity of 86% and 90%, respectively, in an external validation set. In a prospective validation set, sensitivity and specificity were 52% and 98%, respectively, to develop EC within 3 years
Wever, 2023^31^	Case–control	Cervical smear, vaginal swab	EC	320		103	217		qMSP	Methylation of *ADCYAP1*, *BHLHE22*, *CDH13*, *CDO1*, *GALR1*, […^*3*^]	Combining *CDO1*, *GHSR*, and *ZIC1* in vaginal self‐samples results in an AUC of 0.94. Combining *CDH13*, *CDO1*, and *ZIC1* in cervical smears results in an AUC of 0.97
Paracchini, 2023^32^	Case–control	Cervical smear	OC	190	113		77		WGS	Genome‐wide copy number instability	Genomic instability analysis could detect the presence of OC up to 9 years before diagnosis with a sensitivity of 75% and a specificity of 96%
Barrett, 2022^33^	Case–control	Cervical smear	OC, EC	786	47	217	225 (OC), 297 (EC)	*BRCA1* (57) *BRCA2* (53)	Methylation array	Methylation of CpG sites	CpGs methylation identifies OC and EC with an AUC of respectively 0.76 and 0.81. The AUC detection rate of OC in healthy *BRCA1* and *BRCA2* pathogenic variant carriers is 0.62 and 0.54, respectively
Herzog, 2022^34^	Case–control, cohort	Cervical smear, self‐collected vaginal swab, and vaginal swab	EC	562		245	317	Lynch (25)	Methylation array	Methylation of *GYPC* and *ZSCAN12*	In cervical smears, self‐collected vaginal swabs, and vaginal swab samples derived from symptomatic patients, the test detects EC with sensitivities of 97.2%, 90.1%, and 100%, and specificities of 75.8%, 86.7%, and 89.1%, respectively. Additionally, the test identifies 90.8% of EC in samples predating diagnosis up to 1 year
Van Bommel, 2022^35^	Case–control	Cervical smear, vaginal swab	OC	61	29		32		smMIP	Mutation of *ARID1A*, *CTNNB1*, *KRAS*, *MTOR*, *PIK3CA*, […^*4*^]	At least one pathogenic variant was detected in 22% of the vaginal swabs and 38% of the cervical smears. Specificity was 97% and 94%, respectively
Reijnen, 2020^36^	Case–control	Cervical smear	EC	81		50	31		smMIP	Mutation of *ARID1A*, *CTNNB1*, *KRAS*, *MTOR*, *PIK3CA*, […^*4*^]	Mutation panel analysis shows a sensitivity of 78% and specificity of 97%
Sangtani, 2020^37^	Case–control	Tampon	EC	65		38	27		WGS, pyrosequencing, NGS	DNA mutation, copy number and methylation of *RASSF1*, *HTR1B*, and *HOXA9*	The combination of copy number variant analysis in combination with methylation shows a sensitivity of 92% and specificity 86%
Krimmel‐Morrison, 2020^38^	Case–control	Cervical smear	OC	29	8		21	*BRCA1*/*2* (3 in OC group, 9 in control group)	NGS	Mutation of *TP53*	Only three pathogenic *TP53* mutations are determined of eight OC patients (sensitivity 37.8%)
Paracchini, 2020^39^	Cohort	Cervical smear	OC	17	17			*BRCA1* (8) *BRCA2* (2)	ddPCR	Mutation of *TP53*	Retrospective analysis of smears shows a mutation of *TP53* in 64% of smears 6 years prior to diagnosis. *BRCA1/2* are not further distinguished
Arildsen, 2019^40^	Cohort	Cervical smear	OC	15	15				ddPCR	Mutation of *TP53*	A *TP53* mutation is observed in 6 out of 9 (66.7%) diagnostic samples
Liew, 2019^41^	Case–control	Cervical smear	EC	96		46	50		WGS, qMSP	Mutation of *PTEN* and *TP53* Methylation of *BHLHE22*, *CDO1*, *HAND2*, and *TBX5*	Methylation of *BHLHE22* and *CDO1* combined shows sensitivity of 84.8% and specificity of 88.0%. Adding *PTEN/TP53* mutation testing does not improve the detection rate
Wu, 2019^42^	Case–control	Cervical smear	OC	15	5		10		*qMSP*, pyrosequencing	*Methylation of AMPD3*, *NRN1*, *and TBX15*	The methylation of the gene panel shows a sensitivity of 81%, specificity of 84%, and a detection accuracy of 0.91
Chang, 2018^43^	Case–control	Cervical smear	OC, EC	90	30	30	30		qMSP	Methylation of *POU4F3* and *MAGI2*	Methylation of *POU4F3/MAGI2* separately shows a sensitivity of 61% and specificity of 62%/69% in OC and a sensitivity of 83%/90% and specificity 69%/75% in EC
Wang, 2018^44^	Case–control	Cervical smear	OC, EC	1341	245	382	714		NGS	Mutation of *AKT1*, *APC*, *BRAF*, *CDKN2A*, *CTNNB1*, […^*5*^]	A mutated gene is found in 81% and 33% of EC and OC, respectively. Specificity overall is 98.6%. *PTEN* is the most commonly affected gene
Huang, 2017^45^	Case–control	Cervical smear	EC	146		50	96		qMSP	Methylation of *BHLHE22*, *CDO1*, and *CELF4*	Combining 2 of 3 genes reached a sensitivity of 91.8%, and specificity of 95.5%
Bakkum‐Gamez, 2015^46^	Case–control	Tampon	EC	66		38	28		Pyrosequencing	*Methylation of RASSF1*, *HSP2A*, *HOXA9*, *CDH13*, *HAAO*, […^*6*^]	The AUC to detect EC is highest in the genes *HTR1B* (0.82), *RASSF1* (0.75) and *HOXA9* (0.74)
De Strooper, 2014^47^	Cohort	Cervical smear	EC	21		21			qMSP	Methylation of *CADM1*, *MAL*, and *miR124‐2*	76% of women with EC score positive for DNA methylation for at least one of the following genes *CADM1*, *MAL*, and *miR124*‐*2*
Kinde, 2013^27^	Case–control	Cervical smear	OC, EC	60	22	24	14		NGS	Mutation of *PC*, *AKT1*, *BRAF*, *CTNNB1*, *EGFR*, […^*7*^]	Whole gene panel analysis shows a sensitivity of 41% in OC and 100% in EC with a specificity of 100%
Jones, 2013^48^	Case–control	Vaginal swab	EC	48		31	17		Methylation array	Methylation of *HAND2*	Methylation of *HAND2* has an AUC of 0.91 stage 1a disease and 0.97 for more progressed disease
Doufekas, 2013^49^	Case–control	Vaginal swab	EC	78		41	38		Methylation array	Methylation of *GALR1*	Methylation of *GALR1* has a sensitivity of 92.7% and specificity of 78.9%

*Note*: ^1^ *ARID1A*, *KRAS*, *CTNNB1*, *FBXW7*, *KMT2D*, *CHD4*, *CSMD3*, *KCNG4*, *NIPBL*, *PPP2R1A*, *RPL22*, *SETD1B*, *RNF43*, *JAK1*, *FGFR2*, *BCOR*, *DOCK3*, *SPOP*, *SOX17*, *ACVR2A*, *CTCF*, *SOS1*, *LZTR1*, *AP4E1*, *ARH‐GAP35*, *MYOM1*, *BAX*, *WWC3*, *SOX5*, *RACGAP1*, *TNRC6A*, *HOXD8*, *KRIT1*, *PAX2*, *AKT1*, *APC*, *BRAF*, *CDKN2A*, *EGFR*, *MAPK1*, *NRAS*, and *SPEN*; ^2^ *EMX2OS*, *GATA2*, *GDF7*, *JSRP1*, *LRRC8D*, *LRRC34*, *LRRC41*, *LYPLAL1*, *MAX.chr8.145103829*, *MAX. chr10.22624479*, *MAX.chr12.52652301*, *MAX.chr14.103021656*, *MDFI*, *MPZ*, *NBPF8*, *OBSCN*, *SEPT9*, *SFMBT2*, *SQSTM1*, *VILL*, *ZNF90*, *ZNF323*, and *ZNF506*; ^3^ *GHSR*, *HAND2*, *SST*, and *ZIC1*; ^4^ *POLE*, *PTEN*, and *TP53*; ^5^ *EGFR*, *FBXW7*, *FGFR2*, *KRAS*, *MAPK1*, *NRAS*, *PIK3CA*, *PIK3R1*, *POLE*, *PPP2R1A*, *PTEN*, *RNF43*, and *TP53*; ^6^ *GTF2A1*, *ASCL2*, *HTR1B*, *NPY*, *HS3ST2*, *MME*, and *ADCYAP1*; ^7^ *FBXW7*, *KRAS*, *NRAS*, *PIK3CA*, *PPP2R1A*, *PTEN*, and *TP53*.

Abbreviations: AUC, area under the curve; ddPCR, droplet digital polymerase chain reaction; EC, endometrial cancer; NGS, next‐generation sequencing; OC, ovarian cancer; qMSP, quantitative methylation‐specific PCR; smMIP, single‐molecule molecular inversion probes; WGS, whole‐genome sequencing.

Bakkum‐Gamez et al. used a panel of 28 tampon‐based methylated DNA markers to detect EC with promising results in 192 women, including 100 patients with EC. The panel of 28 methylated genes resulted in a 96% specificity and 82% sensitivity (AUC 0.91). A post‐hoc analysis of a panel of three genes yielded AUCs comparable to those of the 28‐panel.^29^

Barrett et al. analyzed CpG island methylation profiles in cervical smears from a large cohort of women (*n* = 786) to detect OC and EC,^33^ resulting in AUCs of 0.76 and 0.81 for respectively. To assess whether this test is also informative in healthy women at high risk of developing OC, the association between methylation and the risk of developing OC in a subgroup of healthy *BRCA1* pathogenic variant carriers was evaluated, resulting in an AUC of 0.62. In a follow‐up validation study, a test using cervical smears for early detection of EC based on DNA methylation at 500 CpG sites was evaluated.^30^ After validating this test in a set of 64 EC patients and 225 controls, resulting in a sensitivity of 86% and a specificity of 90%, the test was validated in a prospective cohort of 150 healthy women who participated in a routine cervical screening program, 54 of whom developed EC within 3 years after sample collection. The sensitivity was substantially lower (52%), most likely because of the long‐term storage of the specimen negatively affecting the signal.

Wever et al. showed an optimal panel of three DNA methylation markers with excellent diagnostic performance for detecting EC in a case–control study of 320 women with an AUC of 0.94 and 0.97 for cervicovaginal self‐samples and cervical swabs, respectively.^31^

Herzog et al. used cervical smears and vaginal swabs to evaluate DNA methylation in the genes *GYPC* and *ZSCAN12* as predictors of EC.^34^ The sensitivity and specificity in a case–control cohort of 137 women with cervical smears (71 EC patients) were 97.2% and 75.8%, respectively, and in another case–control cohort with 251 self–collected vaginal swabs (131 EC patients), they were 90.1% and 86.7%, respectively. In a cohort of 63 women with postmenopausal blood loss (eight EC patients), the vaginal swab test resulted in a sensitivity and specificity of 100% and 89.1%, respectively. They also performed a predictive assessment in 25 Lynch carriers to assess whether the test identifies not only women with cancer, but also women at risk of developing cancer because of genetic predisposition. Cervical smears performed during regular surveillance visits had a sensitivity of 33.3% and a specificity of 100% for detecting EC, with positive and negative predictive values of 50.7% and 92.2%, respectively. Another predictive assessment in a general population of 86 women of whom 32 developed EC, retrospective methylation analysis of cervical smear DNA collected 3 years prior to first diagnosis, revealed a sensitivity and specificity for diagnosing EC of 68.8% and 98.1%, respectively. The sensitivity was 90.8% when samples were analyzed up to 1 year prior to the EC diagnosis.

Wang et al. reported on cancer detection in a large cohort of controls (*n* = 712), OC patients (*n* = 245), EC patients (*n* = 382) and by mutation analysis of 18 genes using cervical smear DNA.^44^ They reported a sensitivity of 33% and 81% in OC and EC patients, respectively, with a specificity of 98.6%.

### 3.2 Microbiome

Seven studies evaluated the microbiome in cervical smears of women with OC and EC (Table 2). Morikawa et al. and Nené et al. demonstrated that the composition of the microbiome differed between patients with OC and healthy controls.^50^, ^52^ Morikawa et al. observed in a relatively small study cohort (*n* = 62) that a diversified, Lactobacilli‐poor microbiome was associated with the presence of OC in premenopausal women.^50^ Nene et al. showed that the prevalence of women with non‐Lactobacilli‐dominated cervicovaginal microbiomes was higher both in patients with OC (*n* = 176) and in women with *BRCA1* pathogenic variants (*n* = 109), compared with age‐matched healthy women and women without *BRCA1* pathogenic variants.^52^

**TABLE 2:** Overview of studies using the cervicovaginal microbiome to detect OC and EC.

Author, year	Study design	Biospecimen	Tumor type	N total	N OC	N EC	N control	High‐risk profile group	Age	Findings
Barczynski, 2023^69^	Case–control	Cervical smear, vaginal swab	EC	96		48 + 21 EH	27		P: 65, 56 C: 54	A PCR‐analysis of the 19 most commonly identified microorganisms was performed. Lacotobacilli was more common in patients with benign lesions, while *M. curtisi* and *D. pneumosintes* was commonly seen in EC
Hakimjavadi, 2022^70^	Case–control	Vaginal swab	EC	61		50	11		P: 60, 62 C: 52	The vaginal microbiome reliably segregates not just benign gynecologic condition from endometrial cancer, but also predicts cancer grade and histology
Morikawa, 2022^50^	Case–control	Cervical smear	OC	62	39		23		P: 56 C: 47, 37	Cervicovaginal microbiota in OC, regardless of the menopausal status, were frequently a diversified community. This implies that a diversified microbiota in premenopausal women could be a biomarking in screening for OC
Gressel, 2021^51^	Case–control	Cervical smear	EC	35		25	10		P: 61, 71 C: 59	The microbial diversity of anatomical ecological niches in postmenopausal women with EC is different compared to benign controls
Nené, 2019^52^	Case–control	Cervical smear	OC	^1^ 360 ^2^ 220	176		184 111	109 (*BRCA1*)		The prevalence of age‐matched women with non‐Lactobacilli‐dominated cervicovaginal microbiomes was higher both in patients with OC and in women with *BRCA1* pathogenic variants who had yet to develop cancer
Walsh, 2019^53^	Case–control	Cervical smear	EC	148		66 + 7 EH	75		P: 62 C: 50	Postmenopausal status, obesity and high vaginal pH (>4.5) were verified risk factor for EC and associated with loss of dominance by Lactobacilli species in the microbiome
Walther‐António, 2016^54^	Case–control	Cervical smear	EC	31		17 + 4 EH	10		P: 64, 54 C: 45	The presence of. *A. vaginae* combined with *Porphyromanas* sp. and high pH is suggestive for EC

*Note*: ^1^Ovarian cancer set (ovarian cancer vs. healthy), ^2^ *BRCA1* set (*BRCA1* mutation without OC vs. *BRCA* wild‐type healthy controls).

Abbreviations: C, controls; EC, endometrial cancer; EH, endometrial hyperplasia; OC, ovarian cancer; P, patients.

A correlation between the occurrence of EC and a high vaginal pH in combination with the presence of some types of bacteria was observed in two other studies.^53^, ^54^

### 3.3 Proteomics

A total of six studies evaluating proteomics in cervicovaginal specimen were included, from which no study included patients with a high‐risk profile (Table 3). Rocconi et al. obtained cervicovaginal fluid samples for liquid chromatography‐mass spectrometry (LC–MS) evaluation. Thirty peptides were detected with a statistical significance for detecting OC. They determined a combination of five proteins that were the best predictors for detecting OC with an AUC of 0.86.^55^

**TABLE 3:** Overview of studies using cervicovaginal proteomics to detect OC and EC.

Author, year	Study design	Biospecimen	Tumor type	N total	N OC	N EC	N control	Biomarker	Findings
Rocconi, 2022^55^	Case–control	Cervical smear	OC	83	33		50	Serine proteinase inhibitor A1, periplakin, profilin1, apolipoprotein A1, and thymosin beta4‐like protein	This five peptide panel model for OC demonstrated a significant increased probability to discriminate OC from controls with an AUC of 0.86
Stockley, 2020^56^	Case–control	Vaginal swab or tampon	OC, EC	125	26	41	58	MCM5	There was no significant difference in levels of vaginal MCM5 biomarker between cancer patients or controls
Cheng, 2019^57^	Case–control	Cervical smear	EC	54		21	33	Phosphocholine, asparagine, and malate	Phosphocholine, asparagine, and malate from cervicovaginal fluid have accuracy of 0.78 in detecting EC
Gasiorowska, 2018^58^	Case–control	Cervical smear	OC	11	8		3	Cysteine‐rich secretory protein 3, fibronectin, and *Ly6*/*PLAUR*	Fibronectin, cysteine‐rich secretory protein 3 and *Ly6*/*PLAUR* are present in 5 of 8 OC patients and absent in controls
Calis, 2016^59^	Case–control	Cervical smear	EC	143		37 (including 7 EIN)	106	CA‐125	Detection of CA‐125 in cervicovaginal secretion has a sensitivity of 78% and specificity of 57% to detect EC or EIN
He, 2011^60^	Case–control	Cervical smear	EC	148		26	122	CA‐125	Levels of CA‐125 were significantly increased in serum, endometrial, and cervicovaginal fluid in women with EC

Abbreviations: AUC, area under the curve; EC, endometrial cancer; EIN, endometrial intraepithelial neoplasia; OC, ovarian cancer.

Calis et al. and He et al. reported that the detection of CA‐125 in cervicovaginal secretion could be used as a potential EC biomarker.^59^, ^60^ In the study of Calis et al., the mean cervicovaginal secretion CA‐125 levels in women with endometrial (pre) malignant pathology (*n* = 37) were significantly higher compared to those wo had other benign gynecological pathologies (*n* = 106).^59^

## 4 DISCUSSION

In this review, an overview of DNA methylation and mutation biomarkers, microbiome, and proteomic biomarkers that are detectable in cervicovaginal smears for the early detection of OC and EC is given. Most studies have studied the field of DNA analysis including both methylation and mutation. Studies generally used a case–control design and reported on OC and EC in the general population. Only a limited number of studies included women with a hereditary high‐risk profile (i.e., *BRCA1/2* pathogenic variant carriers, Lynch syndrome patients and/or PHTS patients). Determining DNA methylation biomarkers in cervicovaginal specimen seems to be the most promising technique for detecting OC and EC early in the general population, and this technique needs to be explored in women with a hereditary elevated risk of developing OC and EC.

DNA methylation techniques have shown to be the most promising for detecting OC and EC, considering the large number of participants included and the relatively high accuracy of the tests. The large heterogeneity of the methylated genes used hampers a clear recommendation of the most relevant genes to detect OC and EC. In a cohort of 780 women, Barrett et al. analyzed CpGs methylation and found AUCs of 0.76 and 0.81 for OC and EC, respectively, based on a set of differentially methylated CpG sites.^33^ Herzog et al. detected EC with a panel of only two methylated genes (*GYPC* and *ZSCAN12*) with a sensitivity and specificity of >90% and 75%, respectively.^34^ This was the only study including Lynch patients resulting in a sensitivity and specificity for EC of 33.3% and 100%, respectively. This remarkable difference in diagnostic accuracy when compared to the general population could be explained by the relatively small number of included Lynch patients (*n* = 25). Wang et al. also showed promising results based on DNA mutation analysis; however, there was a large difference in the ages of EC patients and controls (62 and 34 years, respectively). Given that DNA mutations increase with age, it is difficult to judge whether the outcomes of the test are true discriminators.^61^

With respect to the mutated genes in OC, the most found mutation is *TP53*. In this review, five studies reported on sensitivity of the presence of *TP53* in OC. Sensitivities range. The sensitivities ranged between 33% and 64%.^27^, ^38^, ^39^, ^44^, ^62^ The mutated genes associated with EC are less consistent with multiple genes involved. Reijnen et al. reported on the most common mutations (*n* = 8) with different diagnostic values for detecting EC.^36^ The studies in this review revealed a similar wide range of diagnostic accuracy of the mutated genes in women with EC, illustrating the challenges of using DNA mutations as a screening tool in both OC and EC.^27^, ^36^, ^37^, ^41^, ^44^

Microbiome studies that were performed in this review were generally explorative. Overall, these studies indicate that there might be an association between a Lactobacilli‐poor and high vaginal pH cervicovaginal microbiome and the presence of OC and EC.^50^, ^52^, ^53^, ^54^ However, these findings need to be interpreted with caution, as the mean age of the healthy control group in these studies was significantly lower than that of the cancer group, so the results may have been biased due to age‐specific microbiome differences and menopausal status.^63^ However, in research on cervical precancerous lesions, differences in the cervical microbiome have also been associated with a higher incidence of cervical precancerous lesions.^64^, ^65^ Further work is needed to investigate whether OC‐specific and EC‐specific microbiomes can be identified.

The field of cancer studies has seen a rise in the adoption of proteomics approaches. The use of proteomics‐based technologies such as LC–MS has allowed the discovery of potential biomarkers and patterns of protein expression. Indeed, the genetic and epigenetic changes occurring at the onset of cancer also logically manifest at the protein level; therefore, proteomics is a potentially effective technique. Based on the small sample size and lack of matched controls in published studies, it remains unclear whether proteomics might contribute to the early detection of OC and EC.

Despite including RNA techniques in our search strategy, no articles that used this technique as a diagnostic or screening tool could be identified. Transcriptomics seems to be a yet unexplored field in the detection of OC and EC, although it has been used in the detection of cervical neoplasia. Cervical scrapes can be used for high‐throughput targeted next‐generation RNA sequencing in which human gene expression profiles (with simultaneous detection of genetic mutations in nonsilenced genes) and microbial gene expression profiles can be simultaneously analyzed via high‐throughput methods.^64^, ^66^, ^67^ Further combining these tests with methylation analysis may be worthwhile for women predisposed to OC or EC because of hereditary cancer syndromes.

Overall, the detection rates of EC are greater than the detection rates of OC. This finding can be explained by the diagnostic target organ, the cervix, being in closer proximity to the uterine cavity than to the ovaries and fallopian tubes. This hypothesis is strengthened by the study of Wang et al. compared the detection rate of OC and EC using a cervical smear and a brush inserted into the uterine cavity (Tao brush) in favor of the Tao brush.^44^ Maritschnegg et al. investigated an approach for lavage of the uterine cavity for the early detection of OC.^68^ They demonstrated that cells originating from ovarian tumors are shed and can be retrieved by washing out the uterine cavity. This study successfully detected OC and EC, as well as asymptomatic and clinically undetectable (low CA‐125 and normal ultrasound) OC in a *BRCA1* pathogenic variant carrier, suggesting that this method is a promising tool for the early detection of OC. Since this intra‐uterine lavage technique is more invasive and less patient friendly than a cervicovaginal smear, this was beyond the scope of the current review. However, these findings support the hypothesis that tumor cells shed from the fallopian tube might enter the uterine cavity and may also be detected in cervicovaginal smears.

This systematic review is the first to provide a clear overview of the various methods and techniques used for the early detection of OC and EC using cervicovaginal smear biomarkers. These data are highly valuable for guiding future studies to further explore techniques and improve the diagnostic accuracy of early diagnosis of OC and EC. Whether these techniques are suitable for high‐risk populations is still unclear due to the low number of studies including high‐risk subgroups. Only five studies reported on the detection of OC and EC in high‐risk profile groups, of which only one reported on Lynch syndrome (*n* = 25).^27^, ^29^, ^34^, ^44^, ^45^

Some further limitations need to be addressed. High heterogeneity was found between studies with a wide range of analyzed biomarkers. This makes it difficult to compare the detection rates of the analyzed genes. Due to this heterogeneity, it was unfortunately not possible to perform a meta‐analysis including all studies separately for OC and EC. The study populations were relatively small, with only eight studies reporting on more than 150 patients. Studies differ in including asymptomatic and symptomatic patients. Moreover, for future clinical implementation, the distinction between screening or early detection is relevant and requires the alignment of inclusion criteria in future studies that might be different for EC (postmenopausal bleeding/germline mutations) and OC (germline mutations).

In summary, there is a need for a more patient‐friendly and easily accessible technique to detect OC and EC. This review shows that DNA methylation panel analyses of cervicovaginal smears are feasible techniques as a minimally invasive tool for the early detection of OC and EC in the general population. The future perspectives regarding detection through microbiome and proteomics approaches are currently unclear because of limited data. Implementation in the daily gynecological clinic is hampered by the lack of large prospective validation studies, which are needed due to the large heterogeneity in published studies. In addition, evaluation of cervicovaginal DNA methylation techniques in a hereditary high‐risk population is required to determine the benefit in this specific group of women with increased risk of developing OC and EC, who might benefit most from these innovative approaches.

## AUTHOR CONTRIBUTIONS

**Kevin J. J. Kwinten:** Conceptualization (equal); formal analysis (equal); investigation (equal); methodology (equal); project administration (equal); visualization (equal); writing – original draft (equal); writing – review and editing (equal). **Victor A. Lemain:** Conceptualization (equal); formal analysis (equal); investigation (equal); writing – original draft (equal). **Joanne A. de Hullu:** Writing – review and editing (equal). **William P. J. Leenders:** Writing – review and editing (equal). **Miranda P. Steenbeek:** Writing – review and editing (equal). **Anne M. van Altena:** Supervision (equal); writing – review and editing (equal). **Johanna M. A. Pijnenborg:** Conceptualization (equal); methodology (equal); project administration (equal); supervision (equal); writing – review and editing (equal).

## CONFLICT OF INTEREST STATEMENT

The authors have nothing to disclose for the work under consideration for publication. The authors have nothing to declare.

## Data Availability

None.
