# Correction to Cervicovaginal specimen biomarkers for early detection of ovarian and endometrial cancer: A review

**DOI:** 10.1002/cam4.70107

**Published:** 2024-08-20

Kwinten KJJ, Lemain VA, de Hullu JA, Leenders WPJ, Steenbeek MP, van Altena AM, Pijnenborg JMA Cervicovaginal specimen biomarkers for early detection of ovarian and endometrial cancer: a review. Cancer Med. 2024;13:e70000. doi:10.1002/cam4.70000

The errors are strictly textual, partly due to the editing process.

1. **In the abstract:**

- Line 3, only the abbreviation for endometrial cancer is given, not for ovarian cancer. The word “a” is missing: developing ovarian or endometrial cancer (EC) due to hereditary > developing ovarian cancer (OC) or endometrial cancer (EC) due to a hereditary.
- Line 5, insert the abbreviation OC: Friendly methods to detect ovarian cancer and EC at an early stage. > friendly methods to detect OC and EC at an early stage.
- Line 8, insert the abbreviation OC: On cervicovaginal specimens (smear, swab, or tampon) intended to detect ovarian on cervicovaginal specimens (smear, swab, or tampon) intended to detect OC
- Line 14, insert the abbreviation OC: It seems to be most promising for detecting ovarian and endometrial cancer at early stages in the > seem to be the most promising for detecting OC and EC at early stages in the

2 **In paragraph 3.1**_**|**_ **DNA methylation and mutation analysis:**

- The font size of the text is larger than the rest of the paragraph, starting at ‘Barrett et al. analyzed CpG island …’

3 **In paragraph 3.3**_**|**_ **Proteomics:**

- There is missing a “h” in word “wo” in the line before the last line: significantly higher compared to those wo had other benign > significantly higher compared to those who had other benign

4 **In the discussion:**

- The words “sensitivities range” are double in the paragraph staring with “with respect to…” and need to be deleted: With respect to the mutated genes in OC, the most found mutation is *TP53*. In this review, five studies reported on sensitivity of the presence of *TP53* in OC. Sensitivities range. The sensitivities ranged between 33% and 64%.27,38,39,44,62 > With respect to the mutated genes in OC, the most found mutation is *TP53*. In this review, five studies reported on sensitivity of the presence of *TP53* in OC. The sensitivities ranged between 33% and 64%0.27,38,39,44,62

We apologize for this error.

